# A Descriptive Study of Acute Pediatric Poisoning Age 0–12 Years Old Presenting to Pediatric Emergency Department Hospital Tunku Azizah, Malaysia

**DOI:** 10.7759/cureus.34527

**Published:** 2023-02-01

**Authors:** Yoon N Ong

**Affiliations:** 1 Pediatric Emergency Medicine, Hospital Tuanku Azizah, Kuala Lumpur, MYS

**Keywords:** disposition, pharmaceutical agent, asymptomatic, unintentional, acute pediatric poisoning

## Abstract

Introduction: Acute pediatric poisoning poses significant morbidity and mortality to a country. This study looks at the pattern of acute pediatric poisoning in ages 0-12 years old presenting to a pediatric emergency department in a tertiary hospital in Kuala Lumpur.

Method: We performed a retrospective review of acute pediatric poisoning aged 0-12 years old presenting to the pediatric emergency department of Hospital Tunku Azizah Kuala Lumpur from 1st January 2021 to 30th June 2022.

Results: A total of 90 patients were included in this study. The ratio of female to male patients was 2:3. Oral ingestion was the most common route of poisoning. 73% of patients were from 0-5 years old and primarily asymptomatic. Pharmaceutical agents were the most common agent of poisoning-no mortality in this study.

Conclusion: The prognosis of acute pediatric poisoning was good in the 18 months of the study period.

## Introduction

Acute poisoning is defined as exposure to poison within 24 hours through any route, intentionally or unintentionally [1]. Pediatric poisoning poses significant morbidity and mortality to a country and is one of the reasons for presentation to an emergency department.

According to the statistics by WHO, the mortality rate due to poisoning in Malaysia was 0.66 per 100000 population in 2019 [2]. A National Poison Centre Malaysia study from 2006 to 2015 showed that accidental poisoning was common among the age group 0-5 years old. Over-the-counter medications are the top agent causing poisoning in children [3]. A study by Koh et al. in Singapore revealed that more than 60% of the poisoning cases occurred in the age group 0-4 years old and were mainly unintentional. Oral analgesics or antipyretics are the top agents causing poisoning [4]. A similar trend was seen in a study by Boonchooduong et al. in Thailand [5]. In his study, oral medications were the main source of poisoning. However, the annual report by the American Association of Poison Control Centers in 2020 showed that household and personal care products were the main cause of poisoning in children in the United States [6].

Clinical symptoms of poisoning depend on the agent of poisoning. In Taiwan, a study by Lee et al. revealed that almost half of their patients were asymptomatic. In his study, drugs acting on the neurological system and analgesics were the top two causative agents causing poisoning [7-8]. Studies in Riyadh, Qatar, Egypt, and Turkey showed similar findings whereby more than half of the patients were asymptomatic [9-12].

A study done in the largest children’s hospital in Singapore from 2009-2013 showed that nearly 50% of the patients were discharged home [4]. Meanwhile, almost 80% of the patients in Taiwan were sent home [7-8]. In Riyadh, Qatar, Egypt, and Turkey, 49-99% of their patients were discharged home [9-12].

Hospital Tunku Azizah(HTA) is a tertiary referral Women and Children’s Hospital in Malaysia. The pediatric emergency department sees children aged 0-12 years old and occasionally teenagers from age 13-18 years old with chronic medical illness under pediatric clinic follow-ups. A significant number of poisoning occur in the age group 0-12 years old, which is commonly accidental compared to the age 13-18. Therefore, this study is designed to look at poisoning in the age group 0-12 years old with the main purpose of looking at the pattern of pediatric poisoning in a tertiary hospital in Kuala Lumpur, Malaysia.

This data helps plan public education or create health policies for pediatric poisoning prevention. One good example is the introduction of the Poison Prevention Packaging Act by the United States government in 1970. Poison Prevention Packaging Act established special packaging requirements for 21 categories of toxic household substances and medications. Between 1973 and 1978, unintentional ingestion of all drugs by children younger than 5 years old declined by 44%, preventing nearly 200,000 such ingestions, with more than half of that decline occurring in the first year. Concomitant with these new packaging requirements, the mortality of children younger than 5 years from unintentional poisoning by oral prescription drugs decreased abruptly when the law first became effective, then decreased 45% more between 1974 and 1992 [13].

## Materials and methods

This is a single-centered retrospective study of pediatric poisoning cases at Hospital Tunku Azizah, the largest referral center for women and children in Klang Valley, Malaysia, over 18 months from 1st January 2021 to 30th June 2022.

The main objective of this study is to describe the trend of pediatric poisoning cases age 0-12 years old that present to Pediatric Emergency Department, HTA. The study population consisted of all pediatric poisoning patients aged 0-12 years old who presented to HTA for acute poisoning during the study period. Pediatric poisoning cases with missing data and those presented to the emergency department 24 hours after ingestion of the poisoning agent will be excluded. A convenience sampling approach was used in this study.

The records of all patients seen at the Pediatric Emergency Department, HTA, during the study period were captured electronically in the Hospital Information System (HIS), with their diagnoses coded according to the International Classification of Diseases (ICD) classification system. Data such as date of poisoning, research identification number, age group, agent of poisoning, route of poisoning, type of poisoning, clinical effects, and disposition were collected and recorded in a data collection form which was then entered into SPSS version 20. The primary outcome of interest in this study is the clinical effects of poisoning. The clinical effects were classified as symptomatic or asymptomatic. Symptomatic means patients presenting with clinical symptoms such as respiratory, cardiovascular, neurological, gastrointestinal, dermatological, or ocular symptoms, and asymptomatic means no clinical symptoms. The secondary outcome of interest is the disposition of patients with poisoning. Disposition refers to the placement of patients after initial management in the emergency department. These patients can be observed in the observation ward in the pediatric emergency department, discharged home, or admitted to the pediatric or intensive care unit.

Ethical committee approval was obtained from the Malaysia Medical Research and Ethics Committee before the commencement of the study. SPSS version 20 is used to analyze the data comprised of descriptive statistics such as frequencies and chi-Square to determine the association between age group and clinical effects. A value of P<0.05 is considered statistically significant.

## Results

Demographics

From January 2021 till 30th June 2022, 90 patients aged 0-12 presented to the pediatric emergency department of Hospital Tunku Azizah. Out of the 90 patients, there were 36 (40%) females, and 54 (60%) males. Seventy-three were from 0-5 years old, and 17 were from 6-12 years old. Table 1 shows the demographics of pediatric patients presenting for acute poisoning.

**Table 1:** Demographics of pediatric patients presenting to the pediatric emergency department for acute poisoning.

Profiles	No. of patients (%)
Gender	
Male	54 (60.0)
Female	36 (40.0)
Age group	
0 to 5	73 (81.1)
6 to 12	17 (18.9)

Route of exposure

81 (90%) cases occurred via oral ingestion. Ocular exposure accounted for two cases, while bites and stings were seven cases (table 2).

**Table 2:** Routes and types of agents causing acute pediatric poisoning.

Characteristic	No. of patients (%)
Route of poisoning	
Ingestion	81 (90.0)
Bites/stings	7 (7.0)
Ocular	2 (2.2)
Type of agents	
Pharmaceutical agents	
Antipyretic(Paracetamol)	10 (11.1)
Topical agents	8 (8.9)
Antihypertensives	5 (5.6)
Antihistamines	4 (4.4)
Vitamins	3 (3.3)
Hormones	2 (2.2)
Cough syrups	2 (2.2)
Antibiotics	1 (1.1)
Antidepressants	1 (1.1)
Antilipids	1 (1.1)
Anticoagulants	1 (1.1)
Antidiarrheals	1 (1.1)
Household/Personal care products	
Bleach/Liquid detergent/Floor cleaner/	6 (6.7)
dishwasher	
Paint/thinners/glue	5 (5.6)
Hand sanitisers	5 (5.6)
Silica desiccant gel	3 (3.3)
Shampoo/soap	3 (3.3)
Pesticides	2 (2.2)
Dietary supplements	2 (2.2)
Perfumes	1 (1.1)
Mosquito repellent	1 (1.1)
Food poisoning	10 (11.1)
Bites/stings	7 (7.8)
Others	5 (5.6)

Source of poisoning

The most common source of the poisoning was pharmaceutical agents (n=32, 35.6%), followed by household products (n=28, 31.1%) and food poisoning (n=10, 11.1%). Bites and envenomation accounted for 7 cases (7.8%) and others (n=5, 5.6%).

Clinical effects

A significant proportion of cases (n=49) from 0-5 years old were asymptomatic compared to 3 cases from the age group 6-12 years old (table 3). The association between age group and clinical effects was statistically significant (Pearson Chi-Square 13.836, p =0.001).

Reason of poisoning

Eighty-two cases of poisoning were accidental, with 1 case of intentional and 1 side effect (table 3).

Disposition

Half of the cases (n=45, 50%) were admitted to the ward for monitoring, and 27 (30%) were discharged from the pediatric emergency department. Seventeen cases (18.9%) were observed in the observation bay. Only 1 case was admitted to the pediatric intensive care unit (table 3).

**Table 3:** Reasons, clinical effects, and disposition of pediatric patients presenting with acute poisoning.

Characteristic	No. of patients (%)
Reason of poisoning	
Unintentional	82 (97.8)
Intentional	1 (1.1)
Side effect	1 (1.1)
Clinical effects	
Asymptomatic	52 (57.8)
Symptomatic	38 (42.2)
Disposition	
Ward	45 (50.0)
Home	27 (30.0)
Observation	17 (18.9)
Paediatric Intensive Care Unit	1 (1.1)

## Discussion

During the 18 months, 90 cases of pediatric patients aged 0-12 years old presented to the pediatric emergency department of Hospital Tunku Azizah Kuala Lumpur for acute poisoning. These 90 patients accounted for 0.18% of pediatric emergency department attendance. Studies done in Singapore, and Taiwan reported that 0.2% of the total attendance in the emergency department was due to poisoning [4,7].

More than half of the patients (n=54, 60%) were males, 81.1% were from the age group 0-5 years old, and the poisoning was unintentional (n=88, 97.8%). A prior study by Alwan et al., looking at the poisoning trend among children in Malaysia from 2006-2015, showed that poisoning was more common in the age group of 0-5 years old, males, and unintentional. This trend is seen in high-income and middle-income countries [3-15]. This is because children aged 0-5 begin to learn, explore and play. They are very inquisitive and like to mimic adults. According to Piaget’s stages of development, this is known as the pre-operational stage of development, where their thinking is intuitive and non-logical. Therefore, they are prone to unintentional poisoning [16].

The most common route of exposure was oral ingestion, accounting for 90 % of the cases, followed by bites and stings. Studies done in Thailand, Singapore, Riyadh, Qatar, Turkey, and Taiwan reported that oral ingestion was the most common route of poisoning in age less than 5 years old [4-12]. Pharmaceutical agents were the most common poisoning agent, followed by household products. The top two medications causing poisoning in children were paracetamol and topical medications. These medications are readily available over the counter and can be purchased from the supermarket as they are non-controlled. Topical medications such as the axe brand universal oil or herbal medicated oil are commonly used in Malaysia and available in most houses. Axe brand universal oil contains menthol, eucalyptus oil, methyl salicylate, and camphor. It is used to relieve muscular aches. In Malaysia, parents are unaware of the medicated oils' toxicity, so they tend to place them on lower shelves. Other than that, these bottles do not have a child safety mechanism on the lid. The easy availability and accessibility of the medications and the lack of storage knowledge lead to poisoning. This contrasts with the American Association of Poison Prevention report in 2020, whereby cosmetics/personal care products, followed by household products, were the top two agents causing poisoning [6]. In a study done in Japan from 1987 to 1991, household products were the most common agent causing poisoning in Japan. The household product implicated was a tobacco and attributed to low storage area [14].

Although nearly 60% of the presentations were asymptomatic, half of the patients were admitted to the ward for close monitoring. 30% of the patients were sent home, and only 1 required pediatric intensive care. This is because most Malaysian parents are poor historians. They usually do not bring the sample of an agent of poisoning to the hospital and are unsure of the amount consumed by their children. Emergency doctors find it challenging to identify the amount of the poisoning agents consumed; therefore, they tend to admit those children to the ward for close monitoring. The patient who required pediatric intensive care developed venom-induced coagulopathy from snakebite and required anti-venom in the intensive care ward. No mortality was recorded in this study. Studies done in Singapore, Thailand, Riyadh, Qatar, Turkey, and Taiwan showed that most of their patients were asymptomatic and sent home [4-12]. This suggests that the amount consumed was too minimal to cause any toxicity or symptoms.

This study had its limitations as this was a retrospective study, and data were collected only from a single center.

## Conclusions

From this study, we know that most pediatric patients that presented for acute poisoning were less than 5 years old, the route of poisoning was via oral ingestion, and they were clinically asymptomatic. Firstly, with this data, we can plan for evidenced-based management and investigations for pediatric poisoning cases to reduce hospital access block and overcrowding issues. Secondly, we can advocate for public pediatric poisoning prevention programs targeting the caretakers to store medications at a higher level beyond the reach of small children.
